# Origin, maintenance and spread of antibiotic resistance genes within plasmids and chromosomes of bloodstream isolates of *Escherichia coli*

**DOI:** 10.1099/mgen.0.000353

**Published:** 2020-03-11

**Authors:** Cosmika Goswami, Stephen Fox, Matthew T.G. Holden, Martin Connor, Alistair Leanord, Thomas J. Evans

**Affiliations:** ^1^​ Institute of Infection, Immunity and Inflammation, University of Glasgow, Glasgow, UK; ^2^​ School of Medicine, University of St. Andrews, UK; ^3^​ Dumfries and Galloway Royal Infirmary, Dumfries, UK

**Keywords:** bacteremia, extended spectrum beta-lactamases, horizontal gene transfer

## Abstract

Blood stream invasion by *Escherichia coli* is the commonest cause of bacteremia in the UK and elsewhere with an attributable mortality of about 15–20 %; antibiotic resistance to multiple agents is common in this microbe and is associated with worse outcomes. Genes conferring antimicrobial resistance, and their frequent location on horizontally transferred genetic elements is well-recognised, but the origin of these determinants, and their ability to be maintained and spread within clinically-relevant bacterial populations is unclear. Here, we set out to examine the distribution of antimicrobial resistance genes in chromosomes and plasmids of 16 bloodstream isolates of *E. coli* from patients within Scotland, and how these genes are maintained and spread. Using a combination of short and long-read whole genome sequencing methods, we were able to assemble complete sequences of 44 plasmids, with 16 Inc group F and 20 col plasmids; antibiotic resistance genes located almost exclusively within the F group. *bla*_CTX-M15_ genes had re-arranged in some strains into the chromosome alone (five strains), while others contained plasmid copies alone (two strains). Integrons containing multiple antibiotic genes were widespread in plasmids, notably many with a *dfrA7* gene encoding resistance to trimethoprim, thus linking trimethoprim resistance to the other antibiotic resistance genes within the plasmids. This will allow even narrow spectrum antibiotics such as trimethoprim to act as a selective agent for plasmids containing antibiotic resistance genes mediating much broader resistance, including *bla*C_TX-M15_. To our knowledge, this is the first analysis to provide complete sequence data of chromosomes and plasmids in a collection of pathogenic human bloodstream isolates of *E. coli *. Our findings reveal the interplay between plasmids and integrative and conjugative elements in the maintenance and spread of antibiotic resistance genes within pathogenic *E. coli *.

## Data Summary

All genome data for this study have been deposited in European Nucleotide Archive (ENA). Short-read Illumina sequences were deposited under accession PRJEB12513. The raw FAST5 PacBio sequences and Unicycler assemblies were submitted under the project accession PRJEB33761. The global ST69 isolates with their accession details are in Table S1, (available in the online version of this article).

**Impact Statement**

Autonomously replicating plasmids are important elements determining bacterial resistance to a number of antimicrobial agents. Understanding the origin of these elements, and how they are maintained and spread, is thus crucial in tackling the alarming rise in bacterial antimicrobial resistance. In this paper, we have fully sequenced chromosomes and plasmids from bloodstream isolates of *Escherichia coli *, the commonest cause of bloodstream infection worldwide. Our results identify how antimicrobial resistance genes can be spread by plasmids through a number of mechanisms: direct plasmid transfer by conjugation; horizontal transmission into other plasmids; and transfer into the host chromosome. These results are of broad significance in the fields of bacterial genomics, plasmid biology and antimicrobial resistance. The results advance our knowledge of how plasmids can survive within bacterial hosts that have the ability to produce bloodstream invasion, and how they can spread antimicrobial resistance genes to other bacterial strains. We demonstrate linkage of different antimicrobial resistance genes on plasmids, which will allow co-selection of genes mediating very broad antibiotic resistance even when using a narrow-spectrum agent. Targeting plasmid-mediated antimicrobial resistance thus presents a significant challenge; our results provide a better understanding of how such plasmid-mediated resistance might be tackled in the future.

## Introduction

Resistance to antimicrobial drugs is now widespread in many bacteria, associated with a poorer outcome from infection and increased costs to healthcare systems [1, 2]. In the USA, antibiotic resistant organisms in 2014 were estimated to cause over two million infections and 23 000 deaths [3], while estimates in Europe from 2015 reported 33 000 deaths from such infections, about 75 % of which were healthcare-associated [4]. A report in 2015 chaired by Jim O’Neil estimated that between 2014–2050 the world economy would lose up to 100 trillion US dollars of economic output if the spread of antimicrobial resistance is not checked [5].

Bloodstream invasion by bacteria represents one of the most severe consequences of infection, the commonest isolate being the Gram-negative pathogen *Escherichia coli *, responsible for about one-third of such infections worldwide [6], and showing a steady increase in incidence over the last 10 years [7–9]. Antibiotic resistance in these isolates is widespread and rising. Of particular concern is the rise in incidence of *E. coli* expressing extended spectrum β-lactamases (ESBL) which produce resistance to 3rd generation cephalosporins - in England in 2017 13 % of bloodstream isolates of *E. coli* were resistant to 3rd generation cephalosporins [10], while within Europe the rate was 14.9 % [11]. Similar rates are reported from the USA [12]. Thirty day mortality from bloodstream *E. coli* infections is reported to be about 10–20 % in a number of studies [13–15]. Such infections with ESBL-producing *E. coli* have a worse prognosis [16], particularly if initial therapy is with a third-generation cephalosporin [17]. Rates of resistance to other commonly broad-spectrum antibiotics are also common in *E. coli,* and frequently co-exist; in the European Union in 2017, 6.3 % of *E. coli isolates* had combined resistance to fluoroquinolones, third-generation cephalosporins and aminoglycosides.

The genetic basis of antibiotic resistance is generally well understood. For example, ESBLs are encoded by a number of genes [18], but those of the CTX-M class are some of the most widespread and increasing in incidence [19]. In particular, the CTX-M15 variant is common and geographically widespread [20], particularly in the epidemic ST131 lineage [21]. *bla*_CTX-M_ and other antibiotic-resistance encoding genes are frequently found on plasmids [22]. These autonomously replicating genetic elements can spread through vertical transmission of parent to offspring, but also by horizontal transmission through bacterial conjugation [23]. Plasmids will place a potential selection burden on the cells in which they exist, since replication and translation of plasmid genes will have a negative fitness cost [24]. Thus, antibiotic usage will provide a selective pressure for plasmid maintenance. However, plasmids can survive even in the absence of antibiotic selection, through other mechanisms such as post-segregational killing systems that encode a stable toxin and labile anti-toxin [25], as well as co-evolutionary adaptations in host chromosome and plasmid that reduce fitness costs [26]. Moreover, antibiotic resistance genes can be mobilised from plasmid to chromosome, removing the need for continued antibiotic presence for maintenance [27]. Such genetic mobility also allows plasmids from different microbes to recombine, producing novel plasmids, as well as acquiring new antibiotic-resistance genes.

Horizontal gene transfer and the other factors described in the previous paragraph contribute to the complexity of antimicrobial resistance. Transfer of antibiotic-resistance genes between microbes may increase their spread in pathogenic bacteria. Transfer of these genes from bacteria in farm and other animals may also be significant [28]. Strict control of antibiotic usage has limited the prevalence of some antibiotic-resistant genes, but is not universally the case [29]. Use of narrow-spectrum agents might also limit the generation of resistance to broader spectrum agents, although genetic linkage of determinants of resistance might lead to inadvertent co-selection of resistance to both. Moreover, experimental studies have shown that acquisition of multiple antibiotic resistance genes can offset the fitness cost of either, a genetic interaction known as reciprocal sign epistasis [26, 30]. To what extent these mechanisms are operative in natural communities of pathogenic *E. coli* causing disease in humans is not clear.

In order better to understand the origin, maintenance and spread of antimicrobial resistance determinants within human pathogenic bacteria, we have undertaken a detailed genetic analysis of bloodstream isolates of *E. coli* from patients in Scotland [31]. In this study, we have combined short and long-read genome sequencing of 16 *E. coli* bloodstream isolates of the common ST131 and ST69 lineages to reconstruct the complete chromosomal and plasmid structure of these microbes. A total of 46 plasmids were reconstructed and antibiotic resistance genes in these elements and the corresponding bacterial chromosome analysed. The plasmids were highly heterogeneous with evidence of large amounts of rearrangement by horizontal transfer, both from other *E.coli* strains as well as other Enterobacteriacae. *bla*_CTX-M15_ genes had re-arranged in some strains into the chromosome alone (five strains), while others contained plasmid copies alone (two strains). Integrons containing multiple antibiotic genes were widespread in plasmids, notably many with a *DfrA7* gene encoding resistance to trimethoprim, thus linking trimethoprim resistance to the other antibiotic resistance genes within the plasmids. Our findings show the impact of horizontal spread of antibiotic resistance genes, and mechanisms allowing spread and transmission.

## Methods

### Assembly of sequences

DNA was extracted for short-read Illumina sequencing of 162 genomes at the Wellcome Sanger Centre, UK as described in Goswami *et al*. [31]. For long-read sequencing 16 strains were selected based on higher numbers of ABR genes and plasmid replicons, and was conducted using PacBio SMRT sequencing at the Norwegian Sequencing Centre, University of Oslo, Norway. Two 8-sample multiplex libraries (8-plex) were created and run on separate SMRT cells (PacBio RS2). High quality finished genomes for these 16 genomes were constructed, using both long and short-reads, by hybrid assembly method of UniCycler v4.0.0 (31) under normal mode of assembly, keeping other settings as default. The assembled circular genomes and circular plasmids were then annotated with Prokka v1.11 [32].

### Phylogenetic tree construction

Using the Prokka-annotated genomes, the pan-genomes were investigated for protein clustering using Roary [33] (>95 % amino acid identity). The 44 completed circular plasmid sequences were then extracted and a gene phylogenetic ML tree [34] was built to look into the gene similarity within the plasmids.

### Antibiotic gene and Toxin/Antitoxin pair identification

SRST2 [35] was used on short-reads to determine ABR gene from ARG-Annot database [36], virulence determinants from VirulenceFinder [37] database and plasmid replicon genes from PlasmidFinder [38] database. For identification of these genes in the hybrid assembled contigs, BLASTn (>90 % coverage and >90 % identity) search was performed against them. An inhouse curated database was used for toxin-antitoxin gene identification. Comparison of sequences was done using Artemis genome visualization [39] and EasyFig [40].

### Integron identification

IntegronFinder [41] identified the Class I integron cassettes and the CALIN cassettes within the assemblies with a maximum threshold for the attC sites as 200 bp and a minimum as 40 bp.

### Global ST69 comparisons

**Fig. 4.**
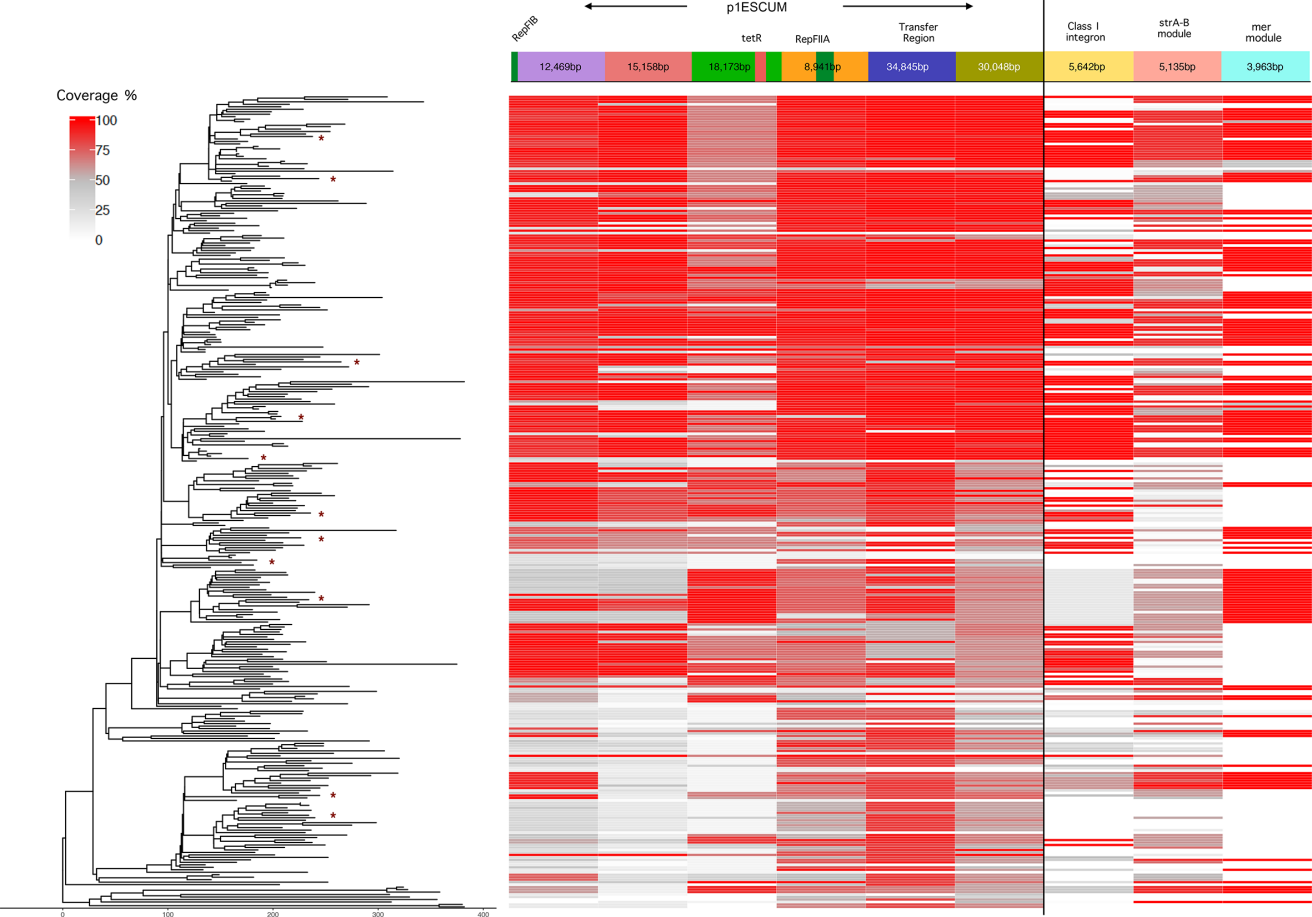
Comparison of Global ST69 Isolates. The UMN026 strain was used as reference genome to map the sequencing reads after masking out the mobile genetic regions. The variants were then identified using VarScan and recombinations were filtered by Gubbins. The midpoint rooted phylogenetic tree is built using RAxML. The x-axis of the tree represents the number of base substitutions along the length of the edges of the tree. The * in the tips of the tree indicates 11 of the 24 ST69 Scottish isolates from [31]; the others were much less related to the global isolates and formed an outlier group on the phylogenetic tree and were thus removed for ease of visualisation of the whole ST69 group . The panels to the right show the coverage of reads over p1ESCUM and the various resistance modules indicated to the right.

An additional 328 ST69 isolates were collected from Enterobase v1.1.2; these are listed with their accession numbers in Table S1. The *E. coli* strain UMN026 (Accession NC_011751.1) was used as the reference genome to map all 328 short-read sequences (including 11 isolates from Scotland). The variants were then identified using VarScan [42] and recombination regions were filtered by Gubbins [43]. The midpoint rooted SNP based phylogenetic tree was built using RAxML [34]. *De novo* assembly of the short-read sequences was performed using SPAdes v3.8.1 [44] assembler. To identify plasmid homologous regions within these short-read sequences, p1ESCUM (Accession CU928148.1, 122 301 bp long) plasmid was divided into six contiguous segments based on its homogeneity (>97 % identity) with complete IncF plasmids (Fig. 4). These six segments were blasted (for >90 % identity threshold) against the *de novo* assembled contigs for percentage of coverage of those regions within 328 isolates. The coverage of three gene cassettes (Class I integron, strA-B module and mer module) were also calculated using BLASTn.

### Conjugation

Bacterial conjugation was performed as described by Johnson *et al*. [45]. Briefly, two donor strains (EC0_10 and EC1_72) and a recipient strain, resistant to rifampicin (DH10B), were grown overnight LB broth without antibiotics. Strains were diluted 1 : 100 in fresh LB and grown for 4 h. Donor and recipient strains were mixed at a ratio of 1 : 10, respectively, and incubated for 18 h without shaking at 37 ˚C. The cultures were heavily vortexed before serial dilutions and plating onto LB agar containing; ampicillin (100 µg ml^−1^) or cefotaxime (1 µg ml^−1^) for donor selection, with and without rifampicin (100 µg ml^−1^), for recipient background selection. The strain conjugation combinations were performed in triplicate. Transconjugant and donor colony forming units were determined by serial dilutions and results are expressed as transconjugants per donor cell input. The lower limit of detection was 10^−8^ transconjugants per donor cell; conjugations were repeated three times.

### Mahalanobis distance determination

Mahalanobis distances of the plasmids from their corresponding chromosomes were calculated using the method described by Suzuki *et al*. [46] and inhouse scripts in R v3.5.3. First, the dinucleotide relative frequencies of the chromosomes were calculated, with a moving window of 5 kb length, along the length of each chromosome as well as plasmids. These frequencies were then used to calculate the value of D2 using function ‘Mahalanobis’ under R package stats. This metric is a measure of the similarity between the sequences of plasmids and their hosts, and has been shown to be a reliable indicator of the similarity of plasmids to their long term hosts. The absolute value of the Mahalanobis distance is difficult to interpret as its upper limit is boundless; a more useful comparator is a derived *p* value which is the probability of the observed value of the Mahalanobis value falling within the empirical distribution of Mahalanobis values for 5 kb segments of the bacterial chromosome. A value approaching one shows high similarity between plasmid and chromosome, a low value the converse. The empirical *p* values were evaluated as frequency of number of plasmid fragments that are greater than the mean distance of a plasmid from its corresponding chromosome. Similar dinucleotide compositions between a plasmid and chromosome gives a *p*-value close to one whereas *p*-values close to zero indicate large distances and dissimilar dinucleotide compositions between a plasmid and chromosome.

## Results

### Selection of bloodstream isolates of *E. coli* for plasmid and chromosomal sequencing

We have recently analysed 162 bloodstream isolates from patients within Scotland in the years 2013–2015 [31]. The two commonest sequence types (ST) were ST131 and ST69 comprising 24 and 16% respectively of the total isolates. ST131 was predominantly isolated in healthcare-associated infection while ST69 was more associated with community-acquired cases [31]. We picked 16 of these isolates for further sequencing using single molecule real time sequencing, 12 ST131 and four ST69 isolates. These isolates were chosen based on being representative of the dominant ST populations, and contained a variety of antimicrobial resistance determinants. We selected isolates that short-read sequences indicated contained the gene for CTX-M15, the main extended spectrum β-lactamase in this collection, and a range of plasmid replicons as identified from short-read sequences. In ST131 and ST69, IncF replicons were present in >95 % of the strains from this collection [31]. This is only a small sample from the whole sequenced collection of bloodstream isolates, but we felt would provide insights into the origin, spread and persistence of antimicrobial resistance genes in representative examples of the *E. coli* bloodstream isolates. A maximum likelihood phylogenetic tree based on the core genomes of these isolates is shown in Fig. S1 together with their content of antimicrobial resistance genes identified from short-read sequencing. This shows the close genetic relationship as expected between the isolates from the same ST group.

We were able to complete plasmid assemblies for 46 plasmids from these isolates by combining the short (Illumina) and long-reads (PacBio) using the Unicycler pipeline [47]; the details of the isolates and plasmids are shown in Table 1. The identified source of the infection was classed as urine for eight of the 16 isolates. Seven of the isolates were resistant to cefotaxime and thus suspected to harbour an ESBL. For the isolate ECO_56, Unicycler was unable to bridge completely two IncF plasmids: ECO_56_C3 and ECO_56_C4. The contigs from these assemblies are very accurate but they have been omitted from some of the analyses where indicated.

**Table 1.** Summary of the Sequenced Plasmids. Resistance (R) or sensitivity (S) to antibiotics is as shown, with abbreviations as follows: TMP. Trimethoprim; CTX, cefotaxime; Gen, gentamicin; AMC, co-amoxiclav; TZP, piperacillin/tazobactam. Minimum inhibitory concentrations (mg l^–1^) for AMC and TZP are shown in parentheses. Accession number at European Nucleotide Archive

Strain	Plasmid	Inc Group	Inc Subgroup	Length	Source of Infection	ST	TMP	CTX	Gen	AMC	TZP	*bla* _OXA-1_	*bla* _CTX-M-15_	Accession Number
EC0_10					Urine	ST131	R	R	R	R (32)	S (8)	+	+	GCA_902668725
	EC0_10_C2	Others	ColRNAI	112708										GCA_902668725
														
	EC0_10_C3	IncF	IncFII/FIBA	106970										GCA_902668725
														
	EC0_10_C4	Col	ColD	5631										GCA_902668725
														
	EC0_10_C5	Others	na	4082										GCA_902668725
EC0_33					Bile	ST131	R	R	S	S (8)	S (<4)	+	+	GCA_902668635
	EC0_33_C10	Col	ColMG828	1546										GCA_902668635
														
	EC0_33_C3	IncF	IncFII/FIA	117124										GCA_902668635
														
	EC0_33_C4	Others	p0111_1	98 727										GCA_902668635
														
	EC0_33_C5	Others	X4/X4TaxC	33 138										GCA_902668635
														
	EC0_33_C6	Col	ColRNAI	3244										GCA_902668635
EC0_4					Urine	ST69	R	S	R	S (8)	S (<4)	−	−	GCA_902668655
	EC0_4_C3	IncF	IncFII/FIBA/P	98 010										GCA_902668655
														
	EC0_4_C4	Col	ColD	5631										GCA_902668655
														
	EC0_4_C5	Col	Col156	5166										GCA_902668655
														
	EC0_4_C6	Col	Col8282	4072										GCA_902668655
EC0_42					Respiratory	ST131	R	S	R	R (16)	S (<4)	−	−	GCA_902668695
	EC0_42_C2	IncF	IncFII/FIBA	144047										GCA_902668695
EC0_56					Unknown	ST131	R	R	R	R (16)	S (8)	+	+	GCA_902668675
	EC0_56_C3*	IncF	IncFII/FIBA/FIA	155220										GCA_902668675
														
	EC0_56_C4*	IncF	IncFII	59 851										GCA_902668675
														
	EC0_56_C5	Others	−	11 371										GCA_902668675
EC0_73					Unknown	ST69	R	R	R	R (32)	S (8)	−	−	GCA_902668625
	EC0_73_C3	IncF	IncFII/FIBA/Q	142696										GCA_902668625
														
	EC0_73_C4	Col	ColD	4409										GCA_902668625
														
	EC0_73_C5	Col	Col8282	4072										GCA_902668625
														
	EC0_73_C8	Col	ColMG828	1549										GCA_902668625
EC0_76					Unknown	ST131	R	S	S	S (2)	S (<4)	+	+	GCA_902668665
	EC0_76_C3	IncF	IncFII/FIBA/FIA	115340										GCA_902668665
														
	EC0_76_C6	Col	ColBS512	2089										GCA_902668665
EC1_20					Unknown	ST131	S	S	S	S (4)	S (<4)	−	−	GCA_902668595
	EC1_20_C2	IncF	IncFII/FIBA/FIA	50 894										GCA_902668595
														
	EC1_20_C3	Col	ColRNAI	5631										GCA_902668595
														
	EC1_20_C4	Col	Col8282	4082										GCA_902668595
EC1_25					Urine	ST131	S	S	S	R (32)	S (8)	+	−	GCA_902668705
	EC1_25_C2	IncF	IncFII/FIA	132945										GCA_902668705
														
	EC1_25_C4	Col	ColK	6888										GCA_902668705
														
	EC1_25_C5	Col	ColMG828	1546										GCA_902668705
EC1_36					Urine	ST69	R	S	S	S (8)	S (<4)	−	−	GCA_902668585
	EC1_36_C2	IncF	IncFII/FIBA/Q	149279										GCA_902668585
														
	EC1_36_C3	Col	Col156	5165										GCA_902668585
														
	EC1_36_C4	Col	Col8282	4072										GCA_902668585
														
	EC1_36_C5	Others	−	2377										GCA_902668585
EC1_5					Urine	ST69	R	S	S	S (4)	S (<4)	−	−	GCA_902668645
	EC1_5_C2	IncF	IncFII/FIBA/Q	147684										GCA_902668645
														
EC1_50					Urine	ST131	R	R	R	R (16)	I (16)	+	+	GCA_902668605
	EC1_50_C2	IncF	IncFII/FIBA/FIA	170727										GCA_902668605
														
EC1_6					Urine	ST131	R	R	R	R (32)	R (128)	+	+	GCA_902668615
	EC1_6_C5	IncF	IncFII/FIBA/FIA	75 763										GCA_902668615
														
	EC1_6_C6	Others	X1TaxC	33 703										GCA_902668615
														
	EC1_6_C9	Col	ColBS512	2089										GCA_902668615
EC1_72					Urine	ST131	R	R	R	R (16)	S (8)	+	+	GCA_902668685
	EC1_72_C13	Others	−	1549										GCA_902668685
														
	EC1_72_C15	Col	ColMG828	1459										GCA_902668685
														
	EC1_72_C4	IncF	IncFII/FIA	91 615										GCA_902668685
														
	EC1_72_C5	IncF	IncFII	70 705										GCA_902668685
														
	EC1_72_C7	Col	Col156	5164										GCA_902668685
														
	EC1_72_C9	Col	Col8282	4087										GCA_902668685
EC1_77					Unknown	ST131	R	S	S	S (8)	S (8)	−	−	GCA_902668715
	EC1_77_C2	IncF	IncFII/FIBA	108851										GCA_902668715
EC1_87					Bile	ST131	R	S	R	S (8)	S (<4)	−	−	GCA_902668575
	EC1_87_C5	IncF	IncFII/FIBA/FIA	170376										GCA_902668575

### Phylogenetic tree of plasmid accessory genome, replicon types and antibiotic resistance genes

**Fig. 1.**
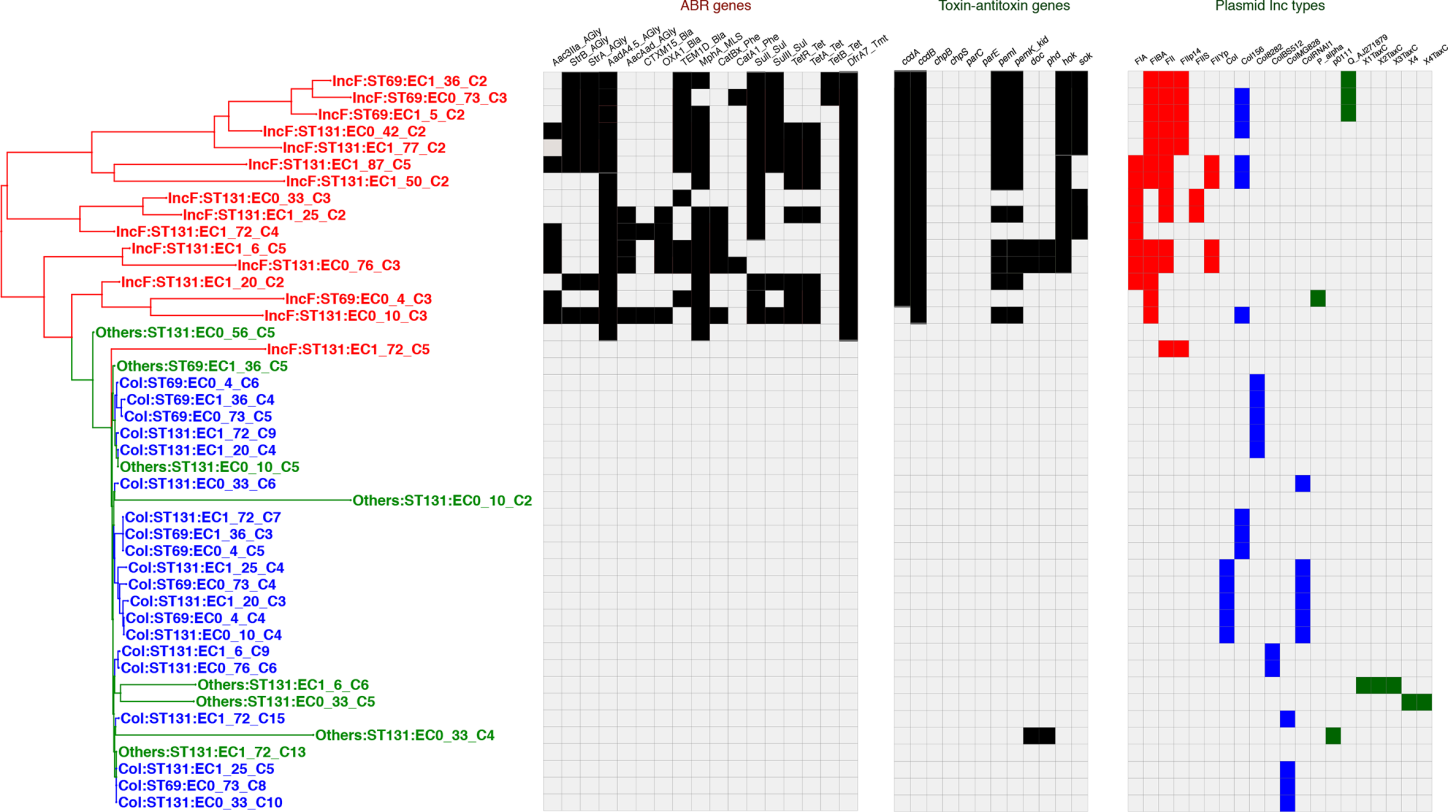
Phylogenetic tree of plasmid isolates. Maximum likelihood phylogenetic tree based on accessory gene content was constructed using RAxML as described in the Methods; bootstrap support values for the tree were greater than 80 %. The tables to the right show antibiotic resistance genes, toxin-antitoxin pairs within the plasmids, and the Inc grouping of each plasmid, based on the database used with PlasmidFinder [38]. IncF groups are coloured red, col type blue and others as green. The resistance determinants were found using ARIBA and the CARD database.

Analysis of the gene content of all the 46 fully reconstructed plasmids revealed a total of 916 genes, 133 of these being shell genes (found in >15 % but <95 % of plasmids); the remaining 783 genes were cloud genes (found in <15 % of plasmids). There were no core genes. Inc F plasmids had the highest percentage of cloud genes (50.0 % of total genes); col and others were much lower (16.7 and 1.24% respectively; Table S2). A phylogenetic tree based on the accessory genome of the plasmids is shown in Fig. 1, together with the replicon types of the plasmids, antibiotic resistance genes, and toxin/antitoxin pairs. Comparison of the different plasmid groups shows that the IncF plasmids were more divergent than the col-type plasmids. Broadly the plasmids fell into three replicon groups: IncF, col-type and others. The larger IncF group plasmids were more diverse than the col plasmids. Plasmids within the IncF group showed multiple F replicons, a feature previously noted using hybridisation assays [48]; three IncF group plasmids also contained IncQ replicon sequences. Whether these different replicons are all functionally active remains to be shown. However, it is notable that of the 16 IncF plasmids, four have four IncF origins, seven have three, two have two, and three just one. This bias towards multiple IncF replicons suggests a positive selection pressure favouring such multiple replicons. This would tend to restrict entry of other IncF plasmids because of plasmid incompatibility and thus a means of these IncF plasmids from preventing entry of other IncF plasmids into the same bacterial cell. Indeed, all but one (EC1_72) of our sequenced strains contained just one IncF plasmid; EC1_72 contains two IncF plasmids, with different replicons. In contrast, plasmids with col replicons typically have only one replicon; of 20 exclusively col replicon containing plasmids, 15 have just one replicon, a significantly different distribution from the IncF plasmids (*P*<0.001, Chi-squared test).

The presence of antibiotic resistance genes within these plasmids is also shown in Fig. 1. Resistance genes were present almost exclusively within the IncF group; one plasmid of unknown incompatibility group did contain some resistance genes (ECO_56_C5). No col-type plasmids contained antibiotic resistance genes. Also shown in Fig. 1 is the presence of toxin/antitoxin pairs important as part of plasmid maintenance through postsegregational killing mechanisms [49]. Again, these are restricted almost exclusively to the IncF plasmid group.

### IncF Plasmids

**Fig. 2.**
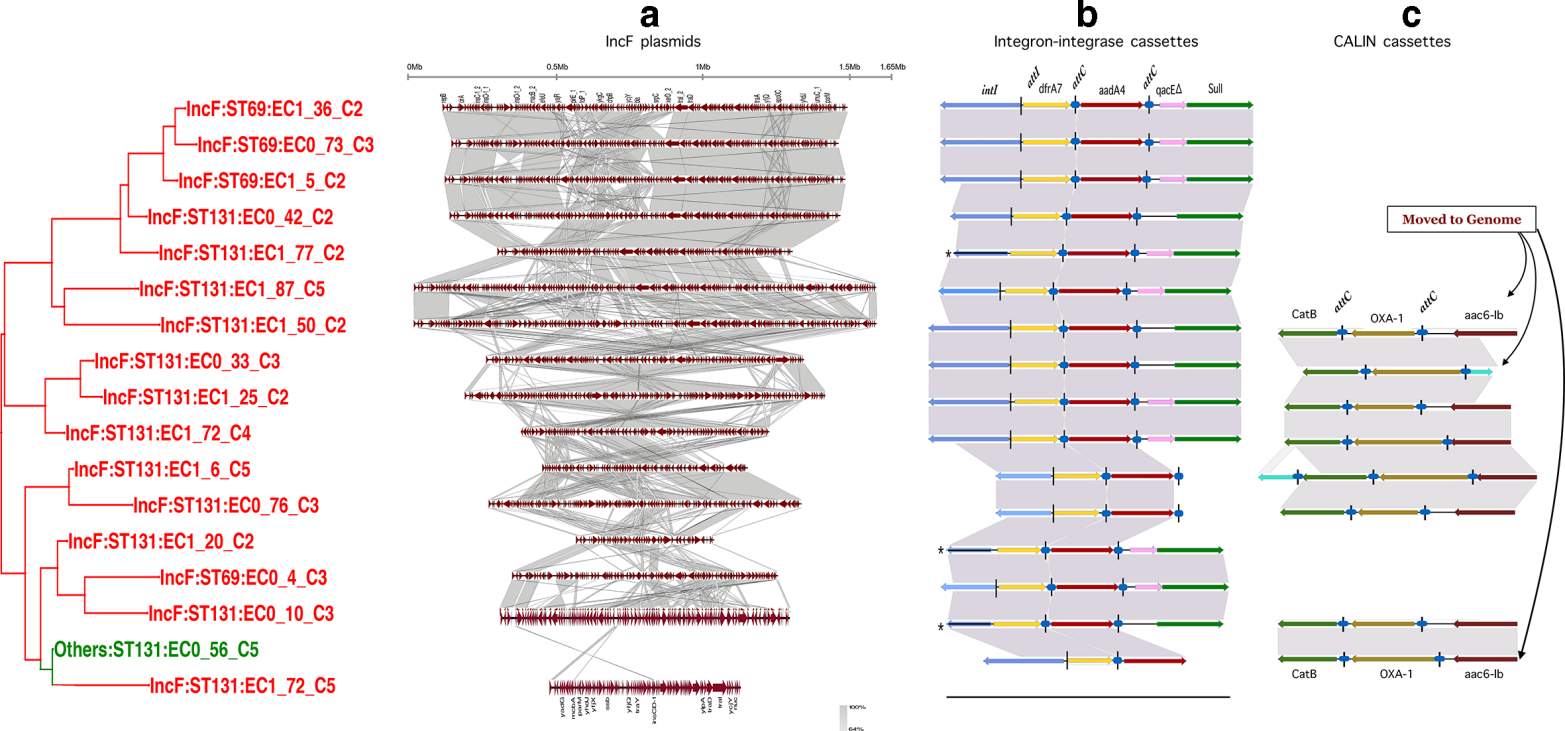
Homology between the IncF Plasmids. The phylogenetic tree of the plasmids is shown to the left. An additional non-IncF plasmid that is closely related is included (ECO_56_C5); only the integron cassettes are shown for this plasmid. The panels to the right show: (a) the outline genetic map of each plasmid with areas of homology between each successive plasmid shaded; the degree of homology is graded as shown by the key; (b) and (c) the identified integron (b) and CALIN (c) cassette elements. The integron recombination sites *attI* and *attC* are as shown.

As the antibiotic resistance genes resided almost exclusively within the IncF plasmid group, we analysed these in more detail. A detailed phylogenetic tree of these plasmids and the regions of similarity is shown in Fig. 2a. Five of these plasmids showed significant overall homology: ECO_73_C3, EC1_36_C2, EC1_5_C2 (all from ST69) and ECO_42_C2 and EC1_77_C2 (both ST131). The remainder showed considerable differences, although with obvious elements of homology, and with marked rearrangements.

### Distribution of Integrons within IncF Plasmids

Given the importance of integrons in mediating and transferring antibiotic resistance, we sought the presence of integrons in all the IncF plasmids sequenced [50–52]. Examination of the IncF plasmid sequences using the program IntegronFinder [41] revealed the presence of the Class I integron discussed above containing the genes *dfrA7* and *aadA4* downstream of the *intI* gene, together with the conserved *sulI* and *qacEΔ* elements of the class I integron [53] in 15 of the IncF plasmids (Fig. 2b).

We also found clusters of attC sites lacking integron-integrases, so-called CALIN elements [41], both within some of the IncF plasmids and in two strains, within the chromosome (Fig. 2c). These all contained the antibiotic resistance genes *bla*_OXA-1_ and *Aac6-Ib*, encoding a beta-lactamase and an aminoglycoside modification enzyme respectively. These genes were tightly linked to the extended spectrum beta lactamase, *bla*_CTX-M15_, and are considered in more detail below.

### Relationship of Plasmids to p1ESCUM

**Fig. 3.**
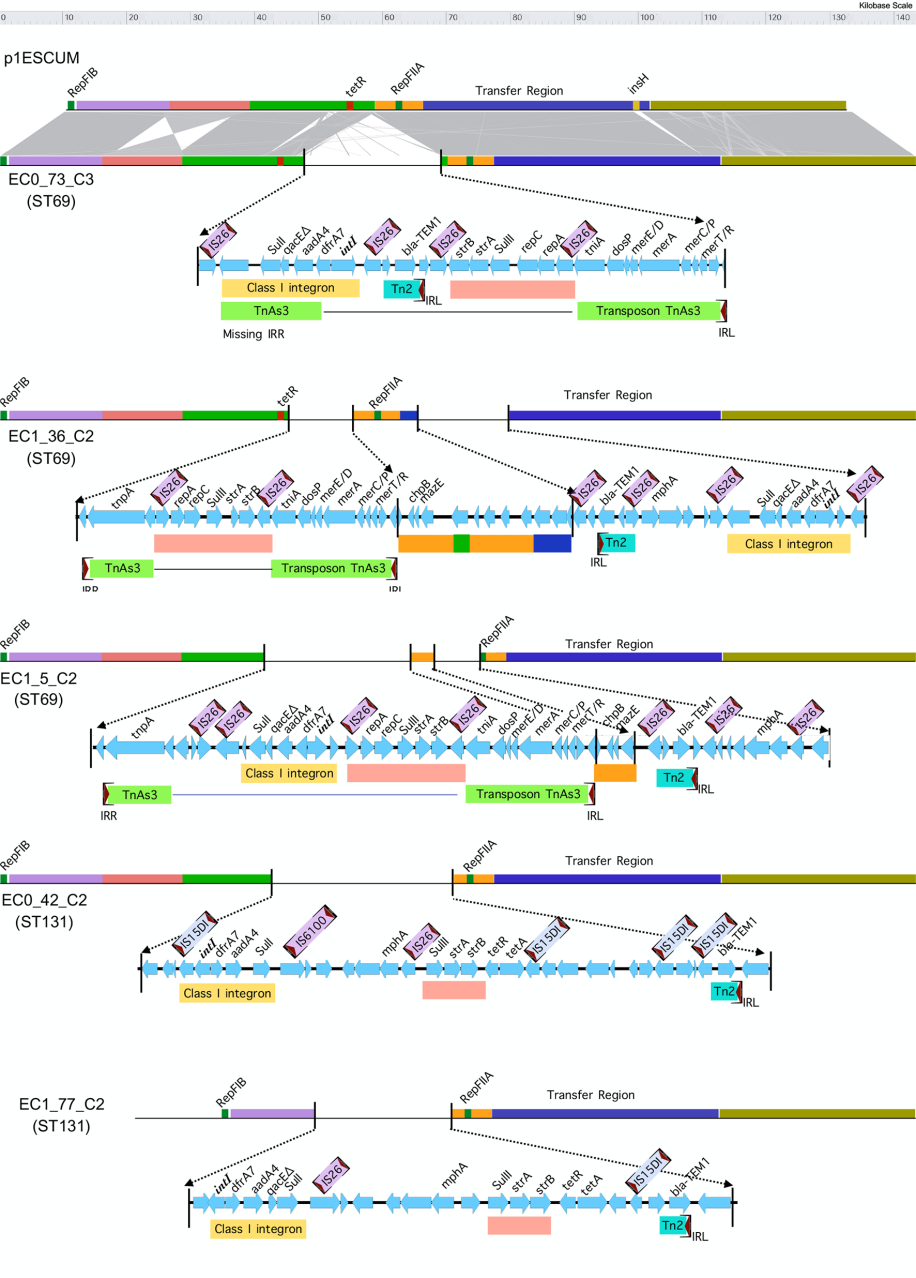
Comparison of 5 IncF Plasmids with p1ESCUM. The indicated plasmids are shown relative to the reference plasmid p1ESCUM. The insertions into the p1ESCUM backbone are highlighted, with genes and mobile elements as shown.

The five plasmids with greatest overall homology (ECO_73_C3, EC1_36_C2, EC1_5_C2 (all from ST69) and ECO_42_C2 and EC1_77_C2 (both ST131)) were analysed in greater detail. Homology search of these related plasmids using blast identified high homology with an *E.coli* plasmid p1ESCUM in strain UMN026 (ST69), isolated in 1999 from a woman with uncomplicated acute cystitis in 1999 in the USA. The percentage identity and coverage are shown in Table S3. Detailed comparison of these plasmids is shown in Fig. 3. Sequences encoding the replicon, type IV conjugal transfer functions and other plasmid backbone features are highly conserved between the different plasmids. p1ESCUM contains very few antibiotic resistance genes. However, the homologous IncF plasmids contain a variety of insertions into the p1ESCUM backbone that contain a variety of antibiotic resistance genes. All the plasmids contain insertions of a Class I integron containing the genes *dfrA7* and *aadA4*, mediating resistance to trimethoprim and streptomycin/spectinomycin respectively [54, 55]. This integron has been found in the IncF plasmid pEK499 from an ST131 *E. coli* [56] and a closely related *dfrA17-aadA5* cassette has been described in a collection of uropathogenic *E. coli* isolated form urine samples of college students in the USA [57]. The ARG-ANNOT data base groups the highly similar *dfrA17* and *dfrA7* together, with a designation *dfrA7*. This resistance determinant was found in 28 % of the total 162 Scottish genomes analysed. It was more prevalent in the ST131 and ST69 strains, at 69 and 50 % respectively, in both cases a significant difference from the total population (two sample z test, *P*<0.001 and<0.05 respectively) . In addition, all the plasmids contain the linked *sulII*/s*trA*/*strB*/ gene cassette, mediating resistance to sulphonamides and streptomycin. This cassette is widespread in mobile genetic elements of a variety of bacteria [58]. Finally, it is notable that all the plasmids contain inserted sequences containing elements of the *mer* operon, a genetic system that encodes proteins responsible for detoxification of Hg^++^ and organomercury compounds to the relatively harmless and volatile Hg^0^ [59, 60]. The relevance of these genes is considered further in the discussion. These different inserted elements are present on a variety of known mobile elements, including transposon TnAs3, initially described in a plasmid of *Aeromonas salmonicida* [61] and also described in plasmids mediating broad antibiotic resistance in strains of *Klebsiella pneumoniae* [62].

### Distribution of p1ESCUM elements and IncF Plasmid Resistance Genes in Global ST69 *E. coli* Isolates

p1ESCUM was isolated from a ST69 *E.coli* strain as were three of the plasmids described above which showed high homology to this plasmid. We wished to determine how widespread this plasmid and derivatives might be in a wider sample of ST69 isolates from different geographical areas, to determine if such homology was also found more widely than strains analysed in our local collection. Long-reads are not available for the vast majority of sequenced bacterial strains, so we were not able to reconstitute complete plasmid sequences. However, we interrogated sequences from 328 ST69 isolates from different geographical areas for the presence of different segments of p1ESCUM and the three resistance gene cassettes identified in plasmids ECO_73_C3, EC1_36_C2, EC1_5_C2 and ECO_42_C2 described above (Table S1,Fig. 4). Conservation of the indicated elements does not imply they are in the original p1ESCUM backbone (or indeed necessarily all together in the same plasmid), as gene rearrangements will lead to reassortment of these sequences into other genetic locations. However, the observed distribution shows widespread presence of homologues of p1ESCUM elements in this collection of ST69 isolates, but not in all. The three resistance cassettes identified above had a more restricted distribution, with clear phylogenetic grouping (Fig. 4). Flux of these elements into or out of these strains has thus occurred on multiple occasions.

### Distribution of CTX-M15 and OXA-1 within IncF Plasmids and Chromosomes

**Fig. 5.**
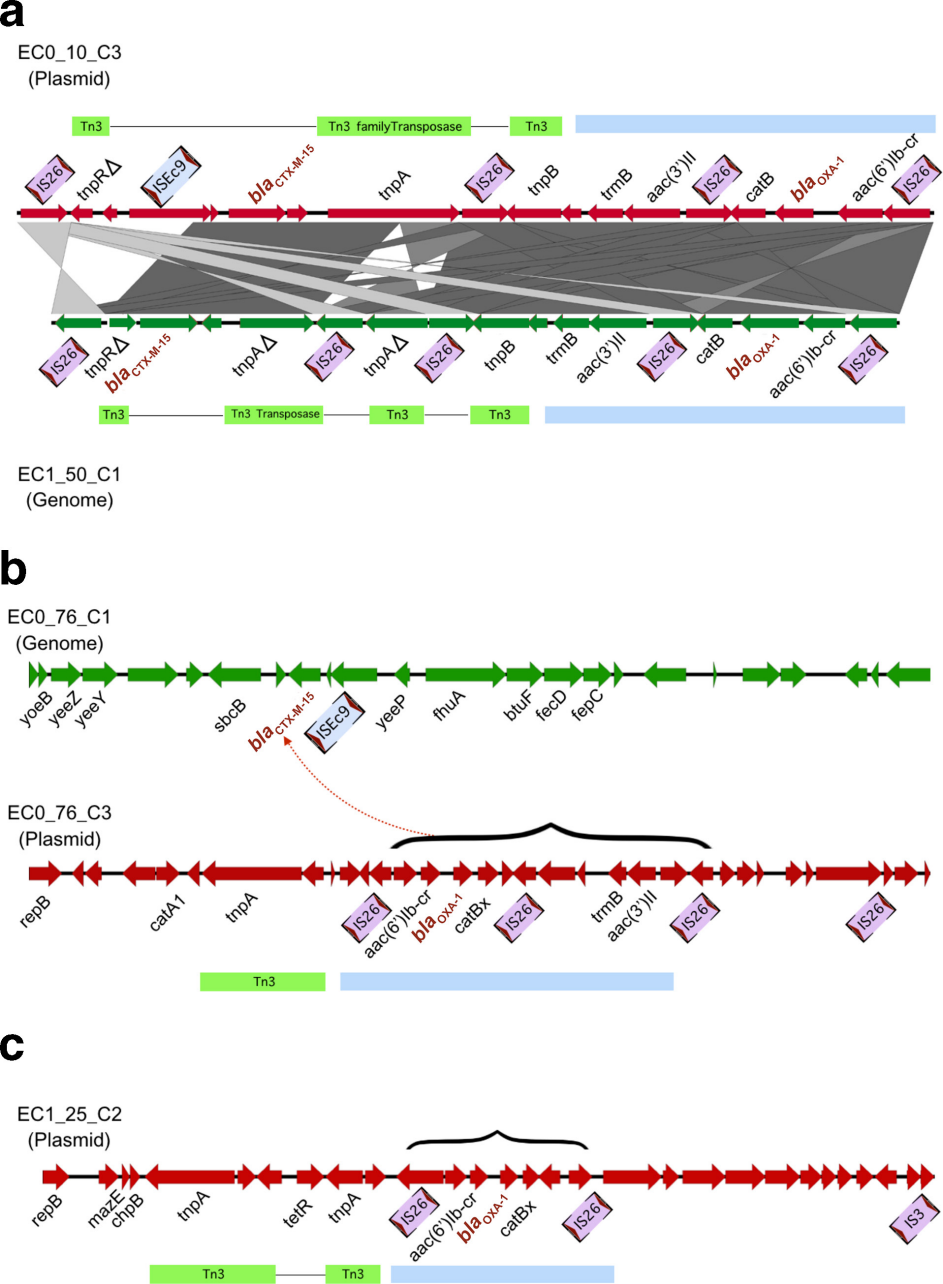
Plasmid and chromosomal positions of *bla*_CTX-M-15_ and *bla*_OXA-1_. (a) shows locations in indicated plasmid and chromosome. Genes and mobile elements are highlighted. The blue block designates the element containing the *bla*_OXA-1_ and *aac(6’)ib-cr* gene. (b) shows location of *bla*_OXA-1_ in chromosome and *bla*_OXA-1_ in plasmid. (c) shows location of *bla*_OXA-1_ alone in plasmid.

Seven of the 16 strains fully sequenced contained the *bla*_CTX-M-15_ encoding an extended spectrum beta-lactamase (Table 2). In two cases this was present in a plasmid, while it was in the chromosome in the remaining five. A number of other antibiotic resistance genes were tightly linked to the *bla*_CTX-M-15_ gene, including the *bla*_OXA-1_ and *aac(6’)Ib* gene contained on a CALIN as described above. The distribution of the *bla*_CTX-M-15_ and *bla*_OXA-1_ genes between plasmids and chromosome is shown in Table 2. Fig. 5a shows a comparison of the plasmid location of this element in EC0_10_C3 and in the chromosome of EC1_50_C1. The element is flanked by IS26 sequences and also contains Tn3 transposase components, suggesting these have been important in its dissemination, a role previously noted for IS26 elements within ST131 *E. coli* [63, 64]. In two cases, the *bla*_CTX-M-15_ gene is located in the chromosome, while the *bla*_OXA-1_ gene is contained on a plasmid; this is shown for the ECO_76 strain in Fig. 5b. Insertion sequence mediated re-arrangements would appear to be instrumental in this rearrangement. In one case, only the *bla*_OXA-1_ gene is present, in plasmid EC1_25_C2; the *bla*_CTX-M-15_ gene is not present in either chromosome or plasmid (Fig. 5c). Again, insertion sequences seem to be responsible for these rearrangements. These data provide a genetic basis for the recently observed co-carriage of *bla*_CTX-M-15_ and *bla*_OXA-1_, mediating resistance to piperacillin/tazobactam and co-amoxiclav, as well as *aac(6’)Ib*, mediating resistance to amikacin and tobramycin [65]. Presence of the *bla*_OXA-1_ gene was associated with an increase in the MIC to co-amoxiclav from 8 to 16 mg l^−1^ and for piperacillin/tazobactam from <4 to 8 mg l^−1^; neither of these shifts were significant between the groups, nor was association between resistance and presence of *bla*_OAX-1_. However, the numbers here are much smaller than those analysed in Livermore *et al*. [65]. One isolate (ECO_73) without *bla*_CTX-M-15_ was resistant to cefotaxime, presumably through upregulation of chromosomal *bla*_AMP-C_. One isolate (ECO_76) with chromosomal *bla*_CTX-M-15_ was sensitive to cefotaxime; there were no sequence alterations in the coding sequence, so this most probably reflects low expression.

**Table 2.** Location of *bla*_CTX-M-15_ and *bla*_OXA-1_ genes within the indicated strains

Strain	*bla*_CTX-M-15_	*bla*_OXA-1_
EC0_10	Plasmid: EC0_10_C3	Plasmid: EC0_10_C3
EC1_72	Plasmid: EC1_72_C4	Plasmid: EC1_72_C4
EC1_50	Chromosome	Chromosome
EC0_33	Chromosome	Chromosome
EC0_56	Chromosome	Chromosome
EC0_76	Chromosome	Plasmid: EC1_76_C3
EC1_6	Chromosome	Plasmid: EC1_6_C5
EC1_25	Absent	Plasmid: EC1_25_C2

**Fig. 6.**
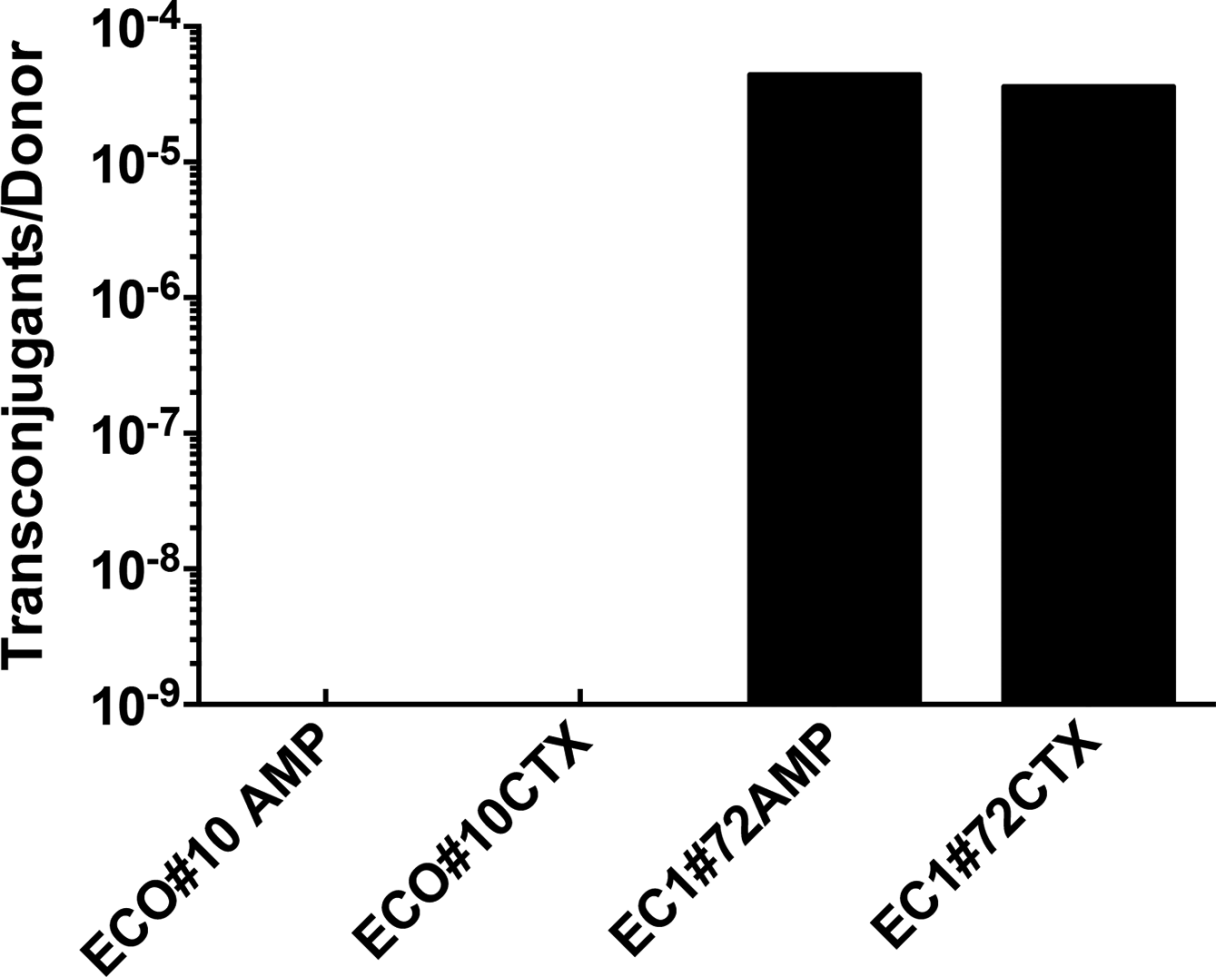
Conjugation efficiency of plasmids. Graph shows the number of transconjugants per donor of transfer from the indicated strains. Columns are means of three determinations; standard error of the means were <0.01 % and are too small to show on the plot. The antibiotic used to select transconjugants is shown next to the strain used: AMP is ampicillin, CTX is cefotaxime. The lower limit of detection was 10^−8^ transconjugants per donor cell.

The relocation of plasmid-borne antibiotic resistance markers such as *bla*_CTX-M-15_ to the chromosome exemplifies aspects of ‘chromosomal imperialism’. Strong evolutionary pressure against plasmid carriage under conditions where plasmid borne genes are not providing benefit to the host will favour chromosomal relocation of potentially advantageous determinants such as genes encoding antibiotic resistance [24, 66]. Thus, survival of plasmids depends strongly on their ability to spread through conjugation [66]. Ten of the 16 IncF plasmids have a conserved specialised type IV secretion apparatus allowing them to be self-transmissible (Fig. S2), supporting the importance of conjugation in plasmid retention in bacterial populations. However, synthesis of the specialised type IV secretion system places a fitness burden on the host, as well as allowing phage predation. Experimental studies have shown that large plasmids eliminate segments encoding the type IV secretion machinery under conditions of continued growth [67], although removing self-transmissibility and thus potentially consigning such plasmids to an evolutionary dead end. Six of the IncF plasmids sequenced here have lost the gene sequences responsible for synthesis of the conjugative apparatus (Fig. S2), suggesting such loss of this portion of a plasmid is not uncommon in naturally occurring plasmid populations. Five of these six plasmids are the only IncF plasmid within their host strain, rendering them unable to spread by conjugation. One plasmid lacking the genes encoding the conjugative apparatus, EC1_72_C4, co-exists in its host with another IncF plasmid, EC1_72_C5 which does contain these genes, potentially allowing donation of transmissibility to the EC1_72_C4 plasmid. To test this, we carried out conjugation assays from the parent strains EC1_72 and ECO_10 (lacking a conjugation apparatus) to a recipient *E. coli* strain, DH10B (Fig. 6). As predicted, conjugal transfer of cefotaxime resistance mediated by the *bla*_CTX-M-15_ gene was successful at high frequency from the EC1_72 strain but below the limit of detectability for the ECO_10 strain.

### Evolutionary History of Plasmids within their Host

In order better to understand the origins of the different plasmids studied here, we wished to estimate how long they had been established in their respective hosts. Over time, ‘foreign’ DNA in a bacterium adopts a very similar composition to its host, a process known as genome amelioration [68]. We calculated the degree of similarity of the dinucleotide frequencies between each plasmid and its bacterial host using the Mahalanobis distance, as described in the Methods [46]. Empirical probability values for the different plasmids sequenced in the current study is shown in Fig. S3; as outlined in the Methods, identity between plasmid and chromosome composition would have a *p* value approaching one. As a reference, we also included two *E. coli* O157 strains (EDL933 and O157:H7 Sakai) that carry the pO157 plasmid, previously included in a study of the relationship between Mahalanobis value and plasmid/chromosome similarity [46]. As has been noted previously [46], there was a significant positive correlation between plasmid length and *p* value, such that the set of small col-origin plasmids were least like their host chromosome, and the bigger IncF plasmids most like their host chromosome. This suggests that the IncF plasmids are likely long-term residents of their hosts, while the smaller col plasmids may be shorter-term residents, potentially colonising a variety of different bacterial hosts. This would need to be verified using a larger set of complete plasmid sequences and their corresponding chromosomes.

## Discussion

The results presented here provide a comprehensive analysis of the plasmids contained within a group of clinical bloodstream isolates of *E. coli* and associated antibiotic resistance genes. Genes conferring antibiotic resistance were almost exclusively located on IncF group plasmids found in all of the isolates, while there were also large numbers of cryptic small plasmids of the col incompatibility group. The IncF plasmids described here thus play a key role in mediating antibiotic resistance in the clinical bloodstream isolates of *E. coli* used in this study. Plasmids require a bacterial host for their continued existence but their existence within bacterial cells potentially places a fitness burden on their host, as significant metabolic activity must be expended for their replication. They have thus evolved mechanisms to resist elimination, such as: toxin/antitoxin pairs to avoid post-segregational loss; possession of genes that confer resistance to environmental stresses such as antibiotics or heavy metals; and the ability to transfer themselves to other hosts. However, blocks to segregational loss are not 100 % effective; resistance genes will only be selected in the face of constant environmental pressure; and conjugation rates do not appear high enough to ensure plasmid survival. Taken together, these blocks have not been thought to be sufficient to explain plasmid persistence, the so-called ‘plasmid paradox’ [69].

Antibiotic resistance will ensure bacterial and hence plasmid survival if the relevant resistance gene is carried on the plasmid. However, movement of the gene to the chromosome will retain the selective advantage of resistance but remove the fitness cost of replicating a large plasmid. The gene for the extended spectrum β-lactamase CTX-M-15 was found in seven of the 16 isolates studied here, but only in two of these was it contained within a plasmid; the rest were found in the chromosome. This is in keeping with the postulated evolutionary pressure for plasmid-borne selectable markers to move to the chromosome. Other genes mediating antibiotic resistance show a similar pattern of spread to the chromosome – the *bla*_OXA-1_-*aac6-1b* cassette carried on CALIN has moved to a chromosomal location in two of the strains. Other determinants, however, are only found on plasmids such as the integron cassette with the *dfrA7* and *aad4* genes. While the selective advantage of antibiotic resistance genes is clear, at least for areas of high usage such as hospitals, it is not clear why there has been retention of the *mer* operon within plasmids that mediates resistance to mercury salts. Mercuric compounds were used extensively in agriculture up to the 1970s, which may have provided a selective advantage for these genes originally for environmental survival of bacteria carrying *mer*-containing plasmids [70, 71]. What selective advantage they now mediate is less clear. One possible explanation for their retention is their co-selection with closely linked genetic elements such as antibiotic resistance genes that confer benefit – such as *bla*_CTX-M-15_ and *bla*_OXA-1_. This may also explain the persistence of the *sulII*/s*trA/strB/* cassette mediating resistance to sulphonamides and streptomycin. Although used extensively in the past, these antibiotic are not used to any great degree currently; again, co-selection with linked genes such as *dfrA7* mediating trimethoprim resistance may play an important role; trimethoprim is used extensively and remains the first-line drug for treatment of uncomplicated urinary tract infections within Scotland. Such co-selection also calls into question the perceived advantages of narrow spectrum antibiotic prescribing, in order to reduce selection for broad spectrum antibiotics. For many of the plasmids described here, use of trimethoprim would confer a selection advantage to *dfrA7* containing plasmids that also mediate resistance to extended spectrum β-lactamases, thus inadvertently selecting for these resistances in addition. Importantly, we have also shown close genetic linkage between *bla*_CTX-M-15_ and *bla*_OXA-1_, explaining the recently observed association between these two determinants, resulting in extended spectrum β-lactamase activity being associated with resistance to co-amoxiclav and reduced sensitivity to piperacillin/tazobactam [65].

The universal presence of IncF plasmids within the 16 isolates described here and in the majority of the 162 isolates previously analysed [31] shows these plasmids are successfully maintained within this population of *E. coli *. The ability to spread by horizontal gene transfer is another key factor in their survival. Ten of the 16 IncF plasmids have a specialized type IV secretion apparatus that can mediate conjugative spread; one *bla*_CTX-M-15_ containing plasmid without this apparatus was successfully transferred by donation from a co-resident self-transmissible plasmid. However, five of the IncF plasmids are not self-transmissible and do not co-exist with a donor plasmid. Long term survival of these plasmids would thus be predicted to be doomed. In addition, these plasmids typically contain more than one IncF replicon, thus preventing uptake of any additional plasmid containing these replicons. Co-evolution of plasmid and host has been shown to ameliorate the negative fitness of plasmid carriage [72], and might contribute to these plasmids survival. Movement of plasmids into new fitter hosts that ‘sweep’ through a bacterial population has also been proposed as a mechanism for continue bacterial survival [66], although this would not be possible for plasmid/host combinations that lack the ability for transfer by conjugation.

In order to better understand how such plasmids bearing antibiotic resistance might spread, we attempted to determine how long they had been present in their hosts and the degree to which they had spread within bacterial populations. Strong homology of three of the IncF plasmids within ST69 strains to the plasmid p1ESCUM allowed us to interrogate a range of available ST69 sequences for the presence of similar elements. Although long-reads are not available for most of these sequences, and hence exact plasmid sequences are not known, we were able to demonstrate widespread possession of p1ESCUM elements within the ST69 lineage, with the antibiotic and mercury resistance elements we identified having a more limited distribution related to phylogenetic origin. This suggests strongly that this plasmid family has been present over significant periods of time and elements have been gained and lost in different sectors of the ST69 lineage. Analysis of the nucleotide similarity between host chromosome and IncF plasmids using the Mahalanobis distance also supports the view that the IncF plasmids are long term hosts of *E. coli* and have not been gained more recently from another bacterial species.

In conclusion, the ability to combine long and short whole genome sequencing reads allows fast and accurate reconstruction of the total plasmid population of bacterial isolates from bloodstream isolates. This has allowed a detailed analysis of the important antibiotic resistance elements present within plasmid and chromosome and how they are spread and retained. The close genetic linkage of many resistance elements has important clinical implications, as co-selection of resistances will occur even when using a narrow spectrum antibiotic, thus rendering antibiotic governance strategies impotent against the spread of resistance to agents such as third generation cephalosporins.

## Data Bibliography

1. Short-read Illumina sequences were deposited under accession PRJEB12513.

2. The raw FAST5 PacBio sequences and Unicycler assemblies were submitted under the project accession PRJEB33761.

3. The global ST69 isolates with their accession details are in Table S1.

## Supplementary Data

Supplementary material 1

Supplementary material 2
