# Supplementary material for: Origin, maintenance and spread of antibiotic resistance genes within plasmids and chromosomes of bloodstream isolates of Escherichia coli
Source: Microb Genom. 2020 Mar 11;6(4):e000353. doi: 10.1099/mgen.0.000353 (PMC7276700; doi:10.1099/mgen.0.000353)
Supplement: Supplementary material 1 [file mgen-6-353-s001.pdf]

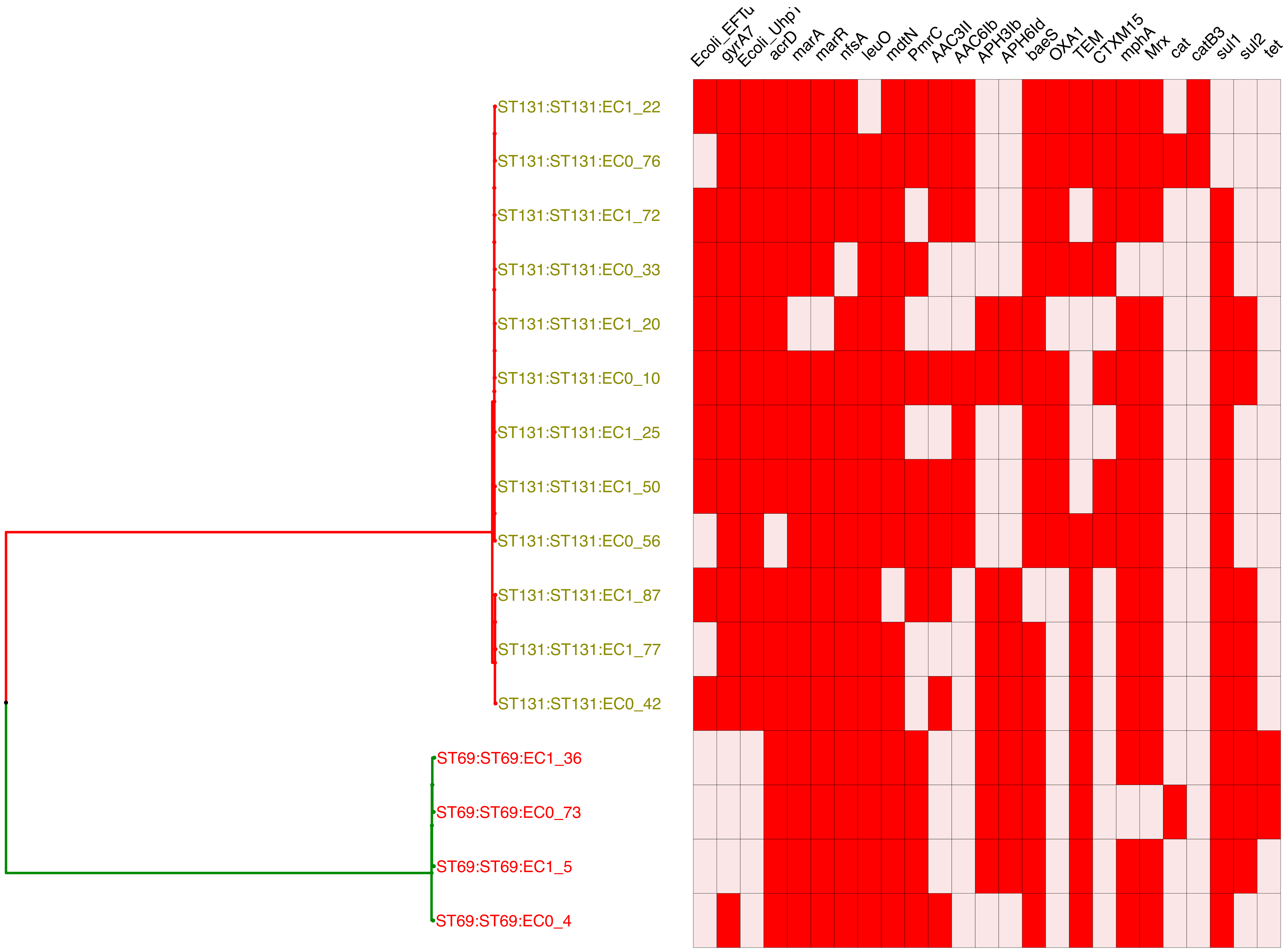

Figure S1. **Maximum likelihood phylogenetic tree of the 16 bloodstream isolates subjected to full sequencing with PacBio.** The table to the right shows the presence or absence of genes mediating antimicrobial resistance identified using the ARG-annot database as described in the Methods.

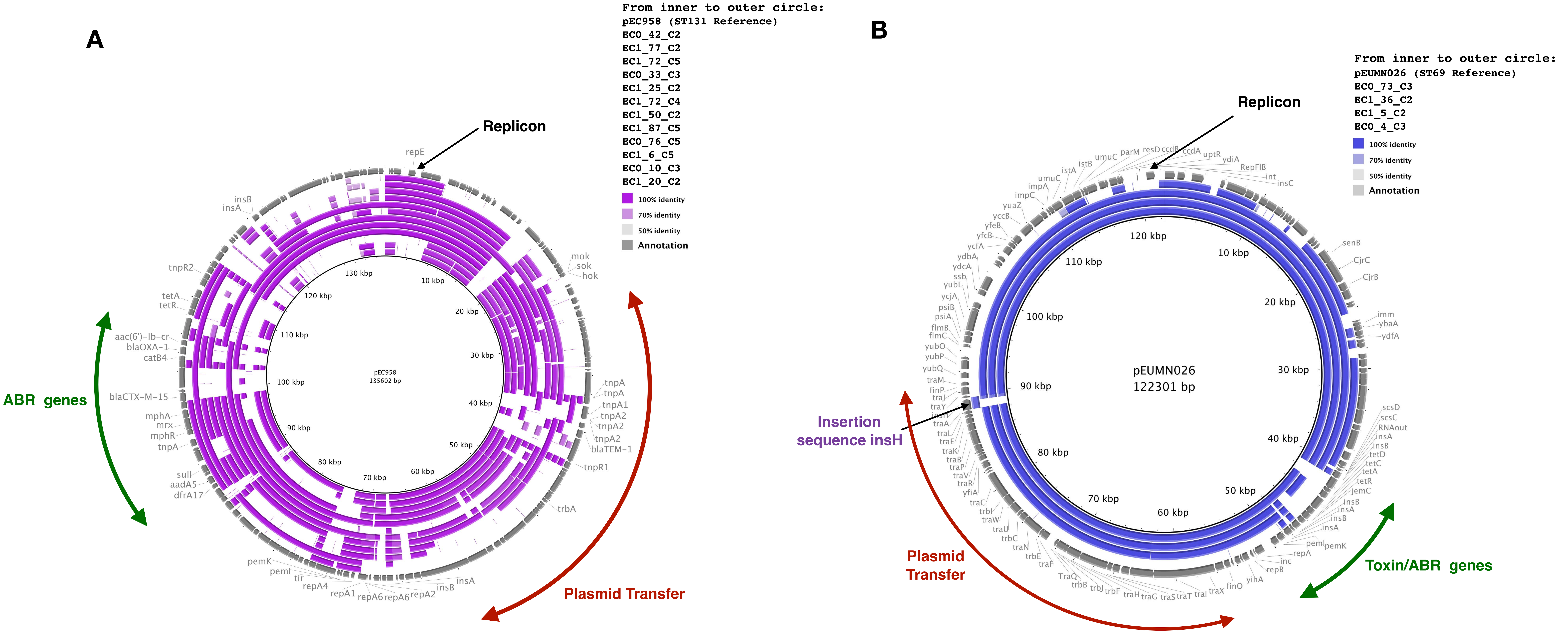

Figure S2. Comparison of different IncF plasmids.
Figure shows the degree of identity of plasmids from ST131 (A) and ST69 (B) aligned to reference pEC958 (A) or pEUMN026 (B). Gene annotations are shown on the outer ring and the various segments of the plasmids annotated as shown.

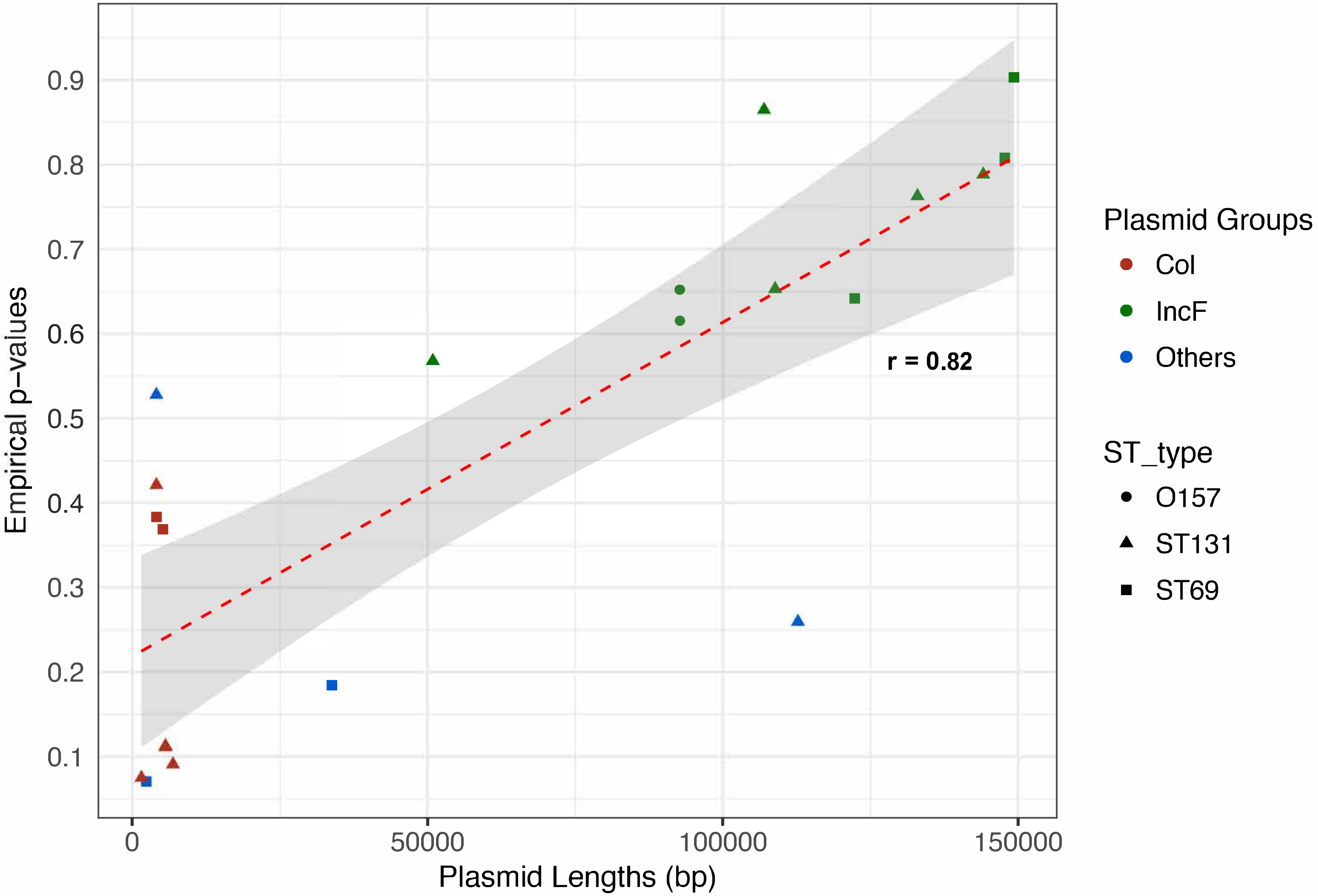

Figure S3. Relationship between empirical Mahalanobis p values and plasmids length. Empirical p value for the comparison of Mahalanobis distance difference between plasmid and chromosome were calculated as outlined in the Methods and plotted versus plasmid length for the indicated plasmids. A smooth linear regression is shown fitted (red dotted line) with 95% confidence intervals shown shaded; the fitted line was significant (p < 10-6), with correlation coefficient of 0.82. 2 IncF plasmids from E. coli strain O157 were included as further reference.

Plasmids	Core genes (99%-100%)	Soft core (95% - 99%)	Shell genes (15-95%)	Cloud genes (0-15%)	Total genes
16 IncF plasmids	0	0	282	279	561
20 Col plasmids	0	0	6	30	36
8 Other plasmids	0	0	5	399	404
All plasmids	0	0	133	783	916

Table S2. Gene sharing statistics between the fully-sequenced plasmids.

Sample	Percent_Identity	Coverage
EC0_73_C3	99.95%	84%
EC1_36_C2	99.93%	79%
EC1_5_C2	99.94%	78%
EC0_42_C2	99.91%	79%
EC1_77_C2	99.97%	78%

Table S3. The percentage identity and coverage of the indicated plasmids to the plasmid p1ESCUM.
